# Supplementary material for: Molecular Characterization and Taxonomic Assignment of Three Phage Isolates from a Collection Infecting Pseudomonas syringae pv. actinidiae and P. syringae pv. phaseolicola from Northern Italy
Source: Viruses. 2021 Oct 15;13(10):2083. doi: 10.3390/v13102083 (PMC8537276; doi:10.3390/v13102083)
Supplement: Supplementary file 1 [file viruses-13-02083-s001.zip › Supplementary_proofs/Table_S6_coverages.docx]

**Table S6**

Coverage of the sequenced
phages

| **Phage** | **Coverage** |
| --- | --- |
| pphageB1 | 600X |
| pphageB2_1 | 709X |
| pphageT1_2 | 664X |
| pphageT2_1 | 655X |
| pphageBV2 | 917X |
| pphageBV4 | 1275X |
| pphageBV7_1 | 983X |
| pphageBV7_2 | 957X |
| psageK4 | 74X |
| psageK4e | 96X |
| psageK9 | 807X |
| psageB2 | 465X |
| psageB1 | 266X |
| psageA1 | 103X |
| psageA2 | 355X |
